# Modeling antibody drug conjugate potential using a granzyme B antibody fusion protein

**DOI:** 10.1186/s12915-024-01860-x

**Published:** 2024-03-14

**Authors:** Trevor S. Anderson, Amanda L. McCormick, Savanna L. Smith, Devin B. Lowe

**Affiliations:** Department of Immunotherapeutics and Biotechnology, Jerry H. Hodge School of Pharmacy, Texas Tech University Health Sciences Center, 1718 Pine Street, Office 1306, Abilene, TX 79601 USA

**Keywords:** Antibody drug conjugate, Antibody fusion protein, Granzyme B

## Abstract

**Background:**

Antibody drug conjugates (ADCs) constitute a promising class of targeted anti-tumor therapeutics that harness the selectivity of monoclonal antibodies with the potency of cytotoxic drugs. ADC development is best suited to initially screening antibody candidates for desired properties that potentiate target cell cytotoxicity. However, validating and producing an optimally designed ADC requires expertise and resources not readily available to certain laboratories.

**Results:**

In this study, we propose a novel approach to help streamline the identification of potential ADC candidates by utilizing a granzyme B (GrB)-based antibody fusion protein (AFP) for preliminary screening. GrB is a non-immunogenic serine protease expressed by immune effector cells such as CD8 + T cells that induces apoptotic activity and can be leveraged for targeted cell killing.

**Conclusions:**

Our innovative model allows critical antibody parameters (including target cell binding, internalization, and cytotoxic potential) to be more reliably evaluated in vitro through the creation of an ADC surrogate. Successful incorporation of this AFP could also significantly expand and enhance ADC development pre-clinically, ultimately leading to the accelerated translation of ADC therapies for patients.

**Supplementary Information:**

The online version contains supplementary material available at 10.1186/s12915-024-01860-x.

## Background

Antibody-based strategies have demonstrated a critical therapeutic role for patients in the modern era of targeted cancer treatments [1]. Since their inception, significant technical advancements have enhanced antibody efficacy by addressing clinical challenges such as immunogenicity, half-life, and targeting capabilities. Such work has also led to the emergence of a specialized category of antibody-based therapeutics, known as antibody drug conjugates (ADCs), which are endowed with direct killing potential by leveraging post-translational modifications to deliver a stable/selective antibody conjugated to a cytotoxic payload [2–4]. The ADC field has witnessed remarkable progress since the development of first-generation molecules in the 1980s, and currently, 13 ADCs have received FDA approval for cancer patients and over 100 molecules are currently being evaluated in clinical trials [5, 6] under, for example, first-line and/or combination scenarios for several types of malignancies [7–11]. Yet, ADC-based approaches still require refinements to address outstanding challenges that include tumor cell targeting, payload potency, patient toxicity, and ADC resistance [6].

Screening lead antibody candidates for ADC potential can be a time-consuming and expensive process. Generally, antibodies targeting tumor-specific/associated extracellular antigens should bind with adequate affinity and minimize off-target effects in vital tissues [12, 13]. Antibodies must also internalize into a target cell via receptor-mediated endocytosis and achieve appropriate accumulation/trafficking for an attached payload that typically disrupts cytoskeletal networks or DNA replication [14, 15]. Once an antibody lead candidate is identified, a standard ADC production route involves generating large quantities of purified antibody, optimizing linker and conjugation chemistries for a payload of interest (e.g., MMAE, SN-38, duocarmycin), and ensuring an otherwise homogenous ADC preparation that maintains effector function against targeted cells. For non-industry laboratories, such expertise and resource-intensive demands can constrain efforts in the ADC field.

There are alternatives that mediate antibody library screening for ADC potential prior to committing to the traditional route of ADC production. One approach involves the manufacturing of small-scale ADC libraries from positive target binders [16]. However, this method is still reliant on performing chemical conjugations/optimizations and purifications of resulting payload-to-antibody ratio compounds [17]. Additionally, the approach may not be readily available for linkers or payloads of interest and may also be confined to in vitro evaluations based on total protein output. An alternative screening path involves the use of a secondary ADC reagent (produced in a separate animal species and conjugated to a payload) that binds antibody candidates [18, 19]. This rapid method relies on the sustained interactions between primary and secondary antibodies to mediate anti-tumoral effects but is limited to in vitro assessments. However, these immune complexes may not accurately reflect important ADC properties such as target binding, internalization/trafficking, and cytotoxic potency.

Antibody-fusion proteins (AFPs) could represent a straightforward alternative to better modeling ADC potential. Previous work with AFPs has attempted to incorporate the molecules as therapeutic options for cancer patients by conjugating plant or bacterial-derived toxins (e.g., saporin, *Pseudomonas* exotoxin A) that abrogate protein synthesis in a targeted cell. These described AFPs are distinct from ADCs though based on cellular mechanisms that induce target cytotoxicity [20] and poor tolerability as a result of toxicities and immunogenicity [21]. However, AFPs could be designed in a manner to more reliably screen for ADC potential by incorporating granzyme B (GrB), a natural product of effector cells such as CD8 + T cells and NK cells during a type-I-induced immune response. GrB represents an intriguing cytotoxic payload for ADC modeling given its non-immunogenicity and ability to induce apoptotic activity in targeted cells through caspase activation [22]. Antibody-based constructs conjugated to GrB have also been extensively studied as cancer targeting therapeutics and ultimately inform on the GrB payload’s ability to deliver in vitro and in vivo cytotoxic effects [23–25].

In this report, we describe for the first time the ability to site-specifically tether an engineered GrB variant (with improved functional characteristics) to a conventional antibody molecule for ADC screening purposes in vitro. This work provides a path for investigators (particularly engaged in protein work) to identify and study further the cytotoxic potential of antibody candidates before producing validated ADCs.

## Results

### Design, expression, and purification of a GrB_mut_-TRA AFP

**Fig. 1:**
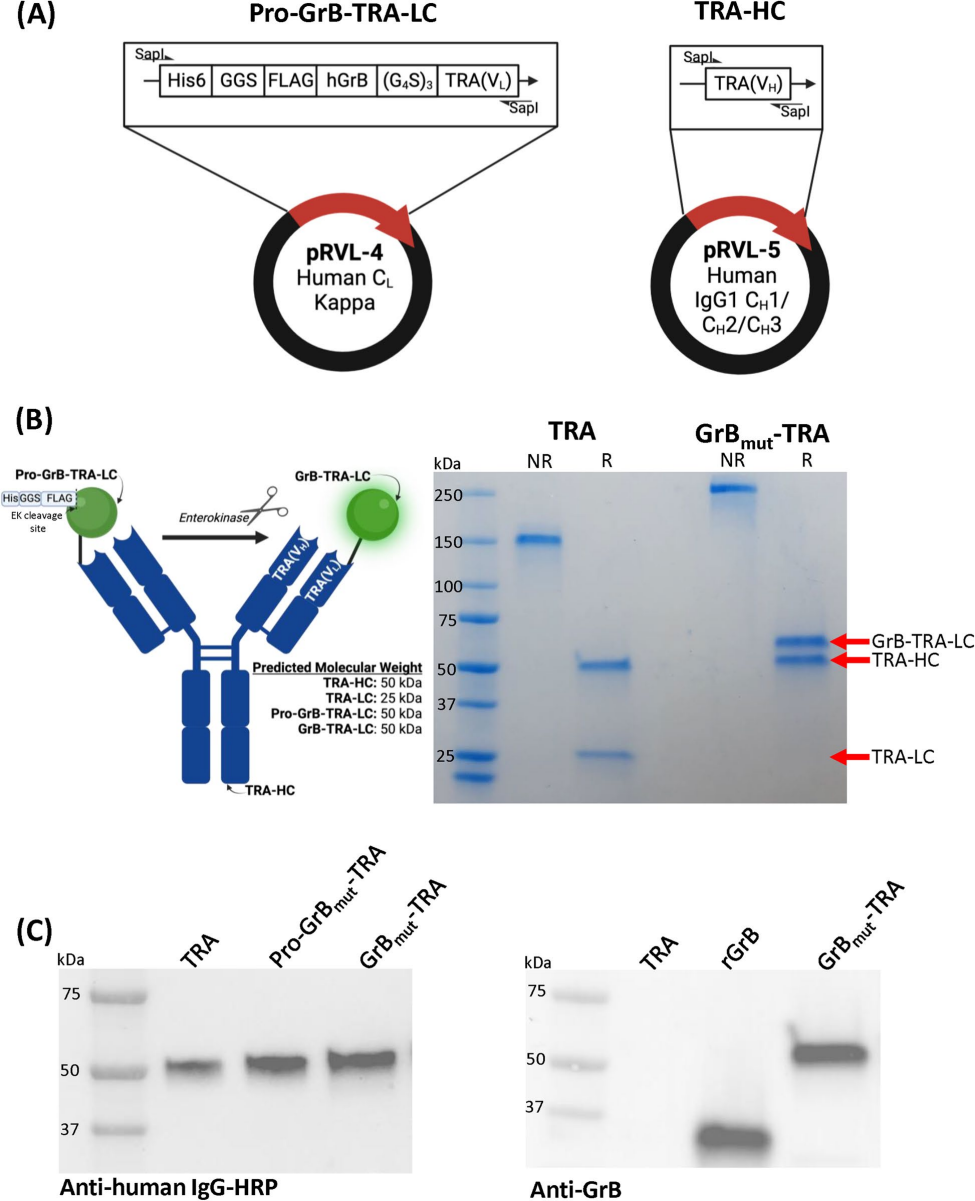
GrB_mut_-TRA construct design and physical characterization. **A** Schematic representation of DNA vectors required for expressing Pro-GrB_mut_-TRA as a human IgG1 antibody in Expi293 cells. Following affinity chromatography purification, EK cleavage was required to generate a catalytically active GrB_mut_-TRA molecule. **B** SDS-PAGE and Coomassie Blue staining of purified TRA and GrB_mut_-TRA under reducing and non-reducing conditions. Arrow insets indicate HC and LC bands for each molecule. **C** Western blot analysis of TRA, Pro-GrB_mut_-TRA, and GrB_mut_-TRA using reagents specific to human IgG and GrB. Abbreviations used: EK, enterokinase; GrB, granzyme B; rGrB, recombinant GrB; GrB_mut_-TRA, catalytically active AFP; HC, heavy chain; LC, light chain; NR, non-reduced; Pro-GrB_mut_-TRA, catalytically inert molecule; R, reduced; TRA, trastuzumab

For modeling purposes, we incorporated the anti-HER2 antibody trastuzumab (TRA) as an AFP in order to demonstrate proof-of-concept and compare efficacy relative to the FDA-approved ADC trastuzumab emtansine (T-DM1). Our AFP design incorporated the pro-form of GrB on the N-terminus of the TRA light chain (LC) to conveniently provide a mature and catalytically active form of GrB following processing/polishing steps (as detailed in the Materials and Methods). In its native pro-form, GrB contains Gly-Glu dipeptide residues on the N-terminus (i.e., designated a pro-peptide) that renders the molecule inert (and non-toxic during expression in cells) and must first be removed by a selective enzyme such as dipeptidyl peptidase I/cathepsin C under physiological settings. In this sense, the resulting N-terminal isoleucine allows GrB to fold into a catalytically active molecule [25]. We, therefore, engineered an experimental pro-peptide that modeled these constraints and also contained an EK cut site and 6X HIS tag to provide controlled release of the pro-peptide following AFP expression/purification and negative selection of AFP variants retaining the pro-form of GrB, respectively (see Fig. 1).

For our ADC screening approach, though, it is critical to utilize a GrB molecule that has been further engineered for improved functional characteristics. To demonstrate this importance, when initially conjugating WT GrB to TRA (designated GrB-TRA) (as similarly described in Fig. 1A), we observed unacceptable AFP targeting properties as a result of non-specific binding. More explicitly, although GrB-TRA interacted with the extracellular domain of HER2 by ELISA and flow cytometry, the AFP also bound to irrelevant proteins, particularly at higher concentrations (Additional file 1: Figures S1A, S1B). These binding effects prevented any discrimination in cytotoxicity between B16 or B16.HER2 target cells (Additional file 1: Figure S1C). Due to several cationic residues, GrB’s overwhelming positive charge likely contributes to electrostatic interactions that manifest in non-specificity as first addressed by others [26–29]. We confirmed such suspicions by disrupting charged interactions with a chelator in our assays. In the presence of EDTA, GrB-TRA interacted specifically with HER2 by ELISA and flow cytometry while non-specific interactions were drastically diminished (Additional file 1: Figures S2A, S2B). However, considering that the enzymatic activity of GrB lessened with increasing concentrations of EDTA (Additional file 1: Figure S2C) and EDTA usage in cell-based in vitro assays would disrupt target cell behavior/viability over several days in culture, we elected to pursue a more robust scenario for AFP modeling by engineering potentially problematic GrB amino acids as described in the Materials and Methods. Overall, we successfully replaced suspected charged residues (R110A, R114A, R116A, K239A, K240A, K243A, K244A), disrupted a putative aggregation site (C210A) (as previously noted [30]), and included a serpin B9 resistance mutation (R201K) (endowing resistance to serine protease scavengers), changes collectively resulting in GrB_mut_. Ultimately, the nature of incorporating a conventional antibody (to more closely mirror FDA approved ADCs) as an AFP with GrB_mut_ has not been described before.

TRA and GrB_mut_-TRA were expressed in Expi293 cells and purified by Protein G affinity chromatography. The GrB_mut_-TRA construct was designed to express an N-terminal pro-peptide (6 × HIS-GGS-FLAG tag) (designated Pro-GrB_mut_-TRA), which prevents GrB_mut_ function (Fig. 1A) and toxicity to Expi293 cells. To activate the AFP, EK (conjugated to 6 × HIS) is admixed to cleave the pro-peptide and release the enzymatically active chain of GrB_mut_. The entire mixture was then subjected to IMAC chromatography to remove unwanted impurities such as uncut Pro-GrB_mut_-TRA, cut pro-peptide, and EK. Additional file 1: Figure S3 demonstrates the integrity of this refining process with our purified GrB_mut_-TRA protein losing HIS and FLAG tag reactivity (compared to Pro-GrB_mut_-TRA) when analyzed by flow cytometry and western blot.

Figure 1B details the SDS-PAGE analysis of TRA and GrB_mut_-TRA that confirmed the purity and predicted molecular weights of the constructs: TRA at 150 kDa (50 kDa heavy chain [31], 25 kDa LC) and GrB_mut_-TRA at 200 kDa (50 kDa HC/LC). The GrB_mut_ component of the fusion protein contains various glycosylation sites, increasing the apparent molecular weight of the GrB_mut_-LC to approximately 60–65 kDa under reducing conditions and 250–260 kDa for the full construct. Western blotting further supported molecular weights as the reduced unmodified HC was evident at 50 kDa for TRA and GrB_mut_-TRA (Fig. 1C) while anti-GrB reactivity demonstrated the AFP’s LC band slightly above 50 kDa (Fig. 1D). These data, overall, demonstrate an ability to reliably produce and purify sufficient quantities of GrB_mut_-TRA with predicted structural characteristics.

### Binding and enzymatic activity of GrB_mut_-TRA

**Fig. 2:**
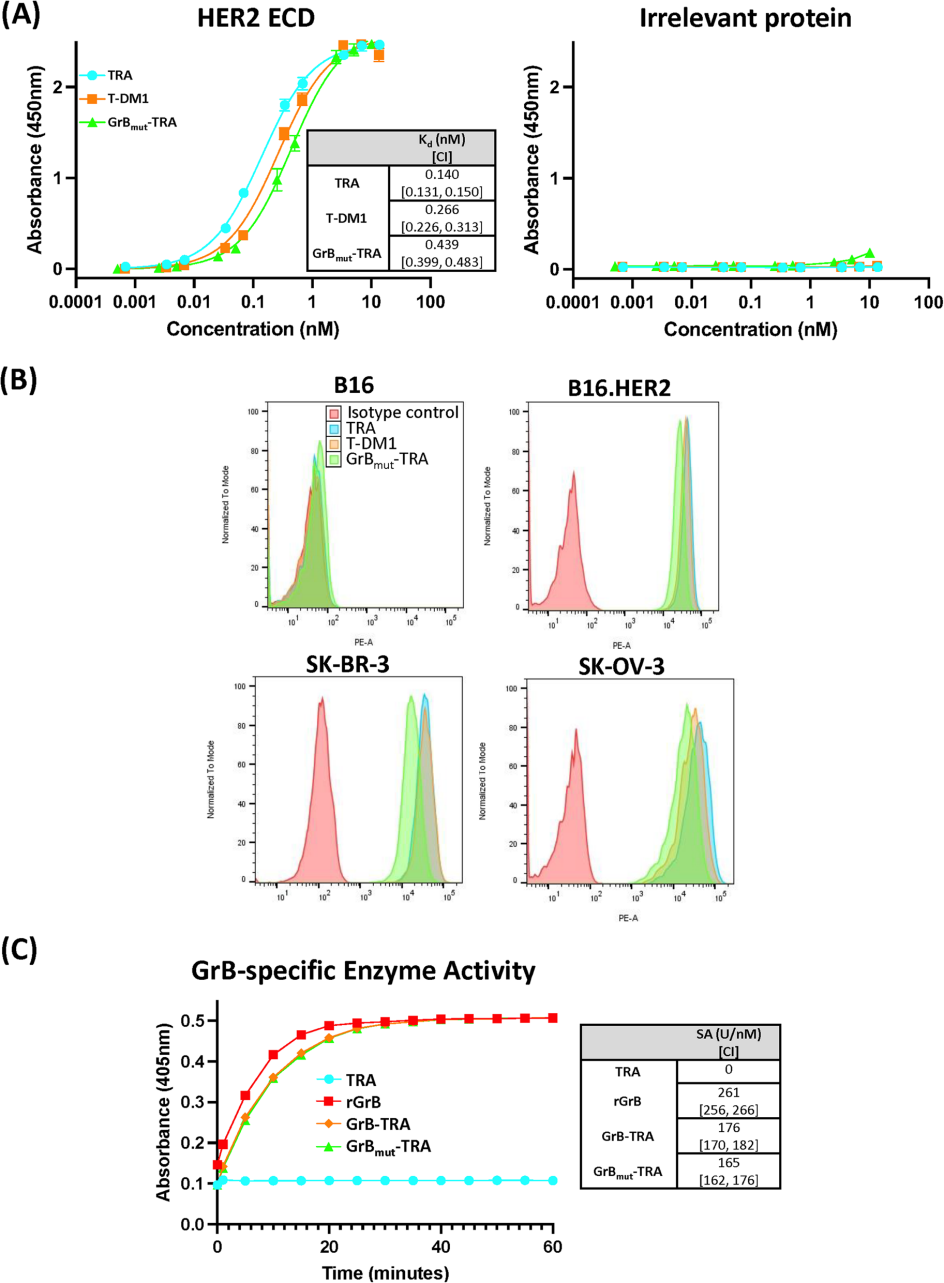
GrB_mut_-TRA target binding and GrB activity. **A** ELISA determination of TRA, T-DM1, and GrB_mut_-TRA binding to immobilized HER2 or irrelevant protein at various concentrations. **B** Assessment of TRA, T-DM1, and GrB_mut_-TRA binding to HER2-expressing (B16, SK-OV-3, SK-BR-3) and non-expressing (B16) cell lines by flow cytometry. **C** Enzymatic activity of GrB was determined through absorbance using a GrB-specific chromogenic substrate (Ac-IEPD-pNA). Abbreviations used: CI, 95% confidence intervals; ECD, extracellular domain; GrB, granzyme B; rGrB, recombinant GrB; GrB-TRA, catalytically active AFP with WT GrB; GrB_mut_-TRA, catalytically active AFP with mutated GrB; SA, specific activity; T-DM1, ado-trastuzumab emtansine; TRA, trastuzumab. Bars ± STDEV. Select results are based on technical replicates of 3 samples per treatment group. Individual data values are provided in supplementary information (Additional file 2)

We next assessed the binding profiles of the AFP relative to TRA and T-DM1. Using indirect ELISA, binding to immobilized HER2 versus an irrelevant protein was initially determined. The observed K_d_ values indicated similar binding profiles between the constructs (TRA 0.140 nM, 95% CI [0.131, 0.150] vs. T-DM1 0.266 nM, 95% CI [0.226, 0.313] vs. GrB_mut_-TRA 0.439 nM, 95% CI [0.399, 0.483]) while non-specific interactions were negligible (Fig. 2A). Flow analysis also demonstrated antibody specificity to membrane-anchored HER2. In scenarios with cell lines expressing HER2 (i.e., B16.HER2, SK-BR-3, and SK-OV-3), GrB_mut_-TRA, TRA, and T-DM1 binding was readily apparent and closely aligned (Fig. 2B). As a control, antibody binding to B16 cells lacking HER2 expression was not observed. A separate experimental AFP created against SIINFEKL/H-2 Kb also demonstrated selective target binding, helping support the generalizability of our approach (Additional file 1: Figure S4). Lastly, we incorporated an absorbance assay to assess the enzymatic activity of GrB_mut_ against a defined caspase substrate. Across conditions that included rGrB, GrB-TRA, GrB_mut_-TRA, and TRA, specific activity was only detected in constructs bearing GrB (Fig. 2C). In separate studies, AFPs not previously subjected to EK digestion were enzymatically inactive (data not shown). Importantly, these results also demonstrated that specific activity of our engineered GrB variant (GrB_mut_) was indistinguishable from WT rGrB (165 U/nM, 95% CI [162, 176] vs 176 U/nM, 95% CI [170, 182], respectively).

### GrB_mut_-TRA target cell internalization and apoptosis

**Fig. 3:**
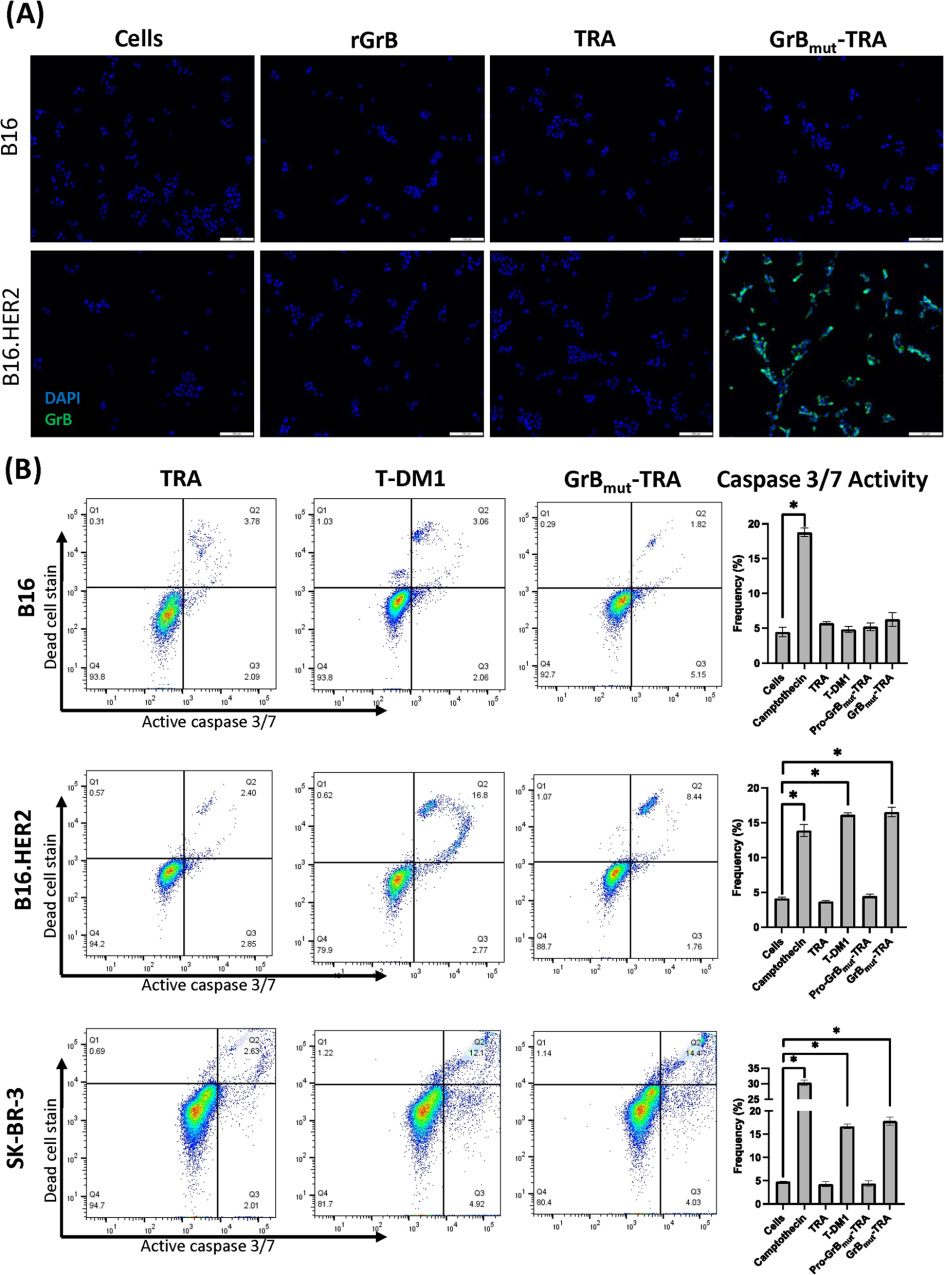
Internalization and apoptosis-inducing effects of GrB_mut_-TRA. **A** B16 and B16.HER2 cells were treated with molar equivalents of rGrB, TRA, or GrB_mut_-TRA before being acid washed, fixed/permeabilized, stained for GrB (green) and nuclei (blue), and assessed by IF. **B** HER2-expressing and non-expressing cells were treated overnight under various conditions that included TRA, T-DM1, or GrB_mut_-TRA. Camptothecin was utilized as a positive control for inducing cell death. All cells were collected and stained with reagents detecting caspase 3/7 and dead cells by flow cytometry. Bar graphs indicate the frequency of caspase 3/7 positive events. Representative histogram plots across treatments are also provided. Abbreviations used: GrB, granzyme B; rGrB, recombinant GrB; GrB_mut_-TRA, catalytically active AFP; Pro-GrB_mut_-TRA, catalytically inert molecule; T-DM1, ado-trastuzumab emtansine; TRA, trastuzumab. *P < 0.05, bars ± STDEV. Select results are based on technical replicates of 2 samples per treatment group. Individual data values are provided in supplementary information (Additional file 2)

GrB_mut_-TRA internalization was subsequently determined by IF using the B16 and B16.HER2 cell lines to document the role of AFP binding HER2. Cells were incubated under various conditions involving activated rGrB alone, TRA, or GrB_mut_-TRA and acid washed to remove surface-bound proteins (confirmed in part by an absence of TRA staining of B16.HER2 cells). As expected, GrB-reactivity was only observed in B16.HER2 cells provided GrB_mut_-TRA, indicating selective target binding as well as internalization of the AFP likely via HER2 receptor-mediated endocytosis (Fig. 3A). The lack of signal in wells treated with an equimolar concentration of rGrB further supports the notion that internalization of the AFP is not mediated by non-specific cellular uptake mechanisms such as pinocytosis. Such data is further corroborated by a report by Cienfuegos and colleagues who documented internalization and cytosolic accumulation of GrB conjugated to a specialized fusion protein [30].

After introduction into a cell, GrB is capable of inducing apoptosis through various intracellular processes, including the direct cleavage of pro-caspase 3 as well as the disruption of general mitochondrial integrity [32]. Therefore, to ensure that internalized GrB potentiated previously documented effects of caspase upregulation [30, 33, 34], we evaluated the caspase 3/7 activation of target cells subjected to the AFP through flow cytometry. B16, B16.HER2, and SK-BR-3 cells were incubated overnight with 500 ng/mL of TRA, T-DM1, or GrB_mut_-TRA. All cells were then collected, stained, and analyzed to determine relative populations bearing caspase-3/7 activity. Interestingly, GrB_mut_-TRA and T-DM1 demonstrated similar levels of caspase induction across HER2-expressing cell lines with minimal activity in the HER2-deficient B16 cell line (Fig. 3B). Identical treatment schemes with TRA, or Pro-Grb_mut_-TRA failed to induce significant caspase-3/7 incidence in cells. These data help confirm the apoptotic-inducing mechanism of internalized GrB_mut_-TRA that was comparable to the microtubule inhibitor payload of T-DM1 [35].

### In vitro cytotoxicity following GrB_mut_-TRA treatment

**Fig. 4:**
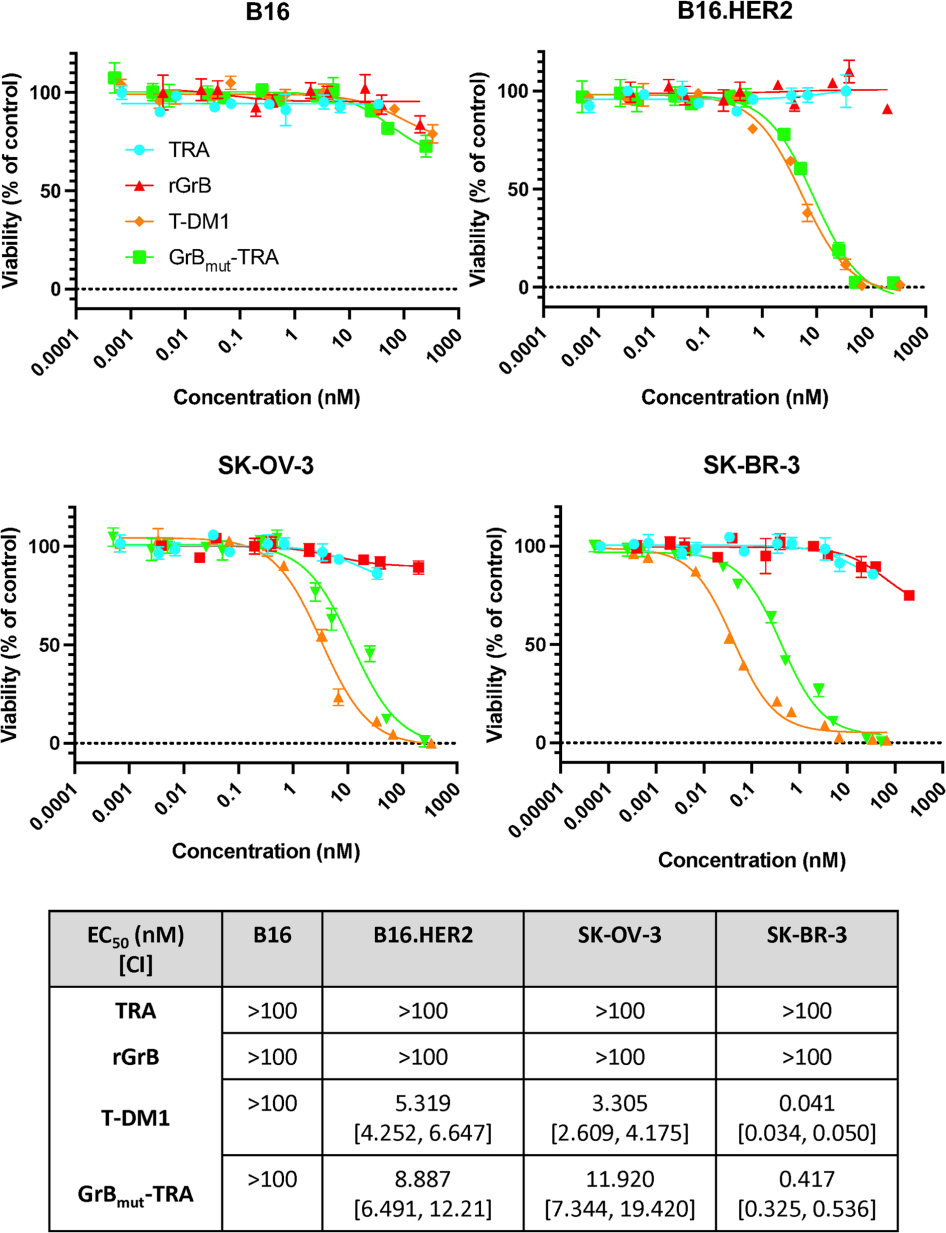
Target cell cytotoxicity following GrB_mut_-TRA treatment. Tumor cell lines deficient in or expressing HER2 were plated overnight and subsequently exposed to various concentrations of rGrB, TRA, T-DM1, or GrB_mut_-TRA. After 48 h, cell viability was determined by crystal violet staining. Target cell viability (%) was calculated based on cells untreated and exposed to 10 μM camptothecin (i.e., maximum cell death). The table inset summarizes EC_50_ values (ng/mL) across tumor cell lines and treatment conditions. Abbreviations used: CI, 95% confidence intervals; GrB, granzyme B; rGrB, recombinant GrB; GrB_mut_-TRA, catalytically active AFP; T-DM1, ado-trastuzumab emtansine; TRA, trastuzumab. Bars ± STDEV. Select results are based on technical replicates of 3 samples per treatment group. Individual data values are provided in supplementary information (Additional file 2)

Finally, target cell cytotoxicity was evaluated in vitro to more conclusively demonstrate AFP-induced killing. Using our panel of tumor cell lines (B16, B16.HER2, SK-OV-3, and SK-BR3), the cytotoxic effects of TRA, T-DM1, and GrB_mut_-TRA were determined after 48 h in culture by crystal violet staining as detailed in the Materials and Methods. Overall, trends in target cell killing were comparable between T-DM1 and GrB_mut_-TRA—although differences existed in general cell line susceptibility between the constructs that could be a result of HER2 copy number on target cells, discrepancies in drug-to-antibody ratios, and/or general sensitivity to payloads (Fig. 4). EC_50_ values for T-DM1 and GrB_mut_-TRA were as follows: B16.HER2: T-DM1—5.319 nM, 95% CI [4.252, 6.647] vs AFP—8.887 nM, 95% CI [6.491, 12.21]; SK-OV-3: T-DM1—3.305 nM, 95% CI [2.609, 4.175] vs AFP—11.920 nM, 95% CI [7.344, 19.420]; and SK-BR-3: T-DM1—0.041 nM, 95% CI [0.034, 0.050] vs AFP—0.417 nM, 95% CI [0.325, 0.536]. Similar to T-DM1 [36, 37], GrB_mut_-TRA’s internalized payload does not easily pass the plasma membrane to inflate cell killing in vitro. Indeed, an advantage to modeling ADC potential with our described AFP is the determination of direct target cell binding and killing without complications from a membrane permeable payload. The relative absence of killing of HER2-negative B16 cells (i.e., > 100 nM) further indicated the targeted nature of our GrB_mut_-TRA to incite cell cytotoxicity. These data do not appear to be a phenomenon relevant only to GrB_mut_-TRA since an AFP targeting the SIINFEKL/H-2 Kb molecule was also capable of selectively inducing cell cytotoxicity in vitro (Additional file 1: Figure S4).

Ultimately, for ADC modeling purposes, our results help establish the credibility of a GrB-conjugated AFP to selectively interact with an antigen of interest, internalize, and induce target cell cytotoxicity.

## Discussion

To date, ADCs have provided patients improved prognoses and represent a prominent fraction of antibody-based therapeutics in development against a variety of malignancies [38]. Yet, producing and authenticating an ADC can be complex and generally requires specialized expertise and resources not readily available for certain laboratories. In vitro screening assays can be useful in assessing monoclonal antibody potential as an ADC prior to engaging in the demands of ADC development. Our use of a particular AFP expands ADC screening potential to better evaluate the ability of an antibody to (i) specifically bind an antigen of interest and address concerns such as on-target/off-target effects, (ii) internalize via receptor-mediated endocytosis, and (iii) achieve sufficient trafficking and accumulation to induce target cell destruction. For our AFP (GrB_mut_-TRA), we incorporated the serine protease GrB, which is a natural component of cytotoxic granules released by immune effector cells such as CD8 + T and NK cells to induce apoptosis in targeted cells through a multi-modal process that involves caspase activation. Additionally, for modeling purposes in vitro, antibody frameworks were mainly based on the well-characterized anti-HER2 antibody TRA although we confirmed major aspects of this work with a TCR-like antibody (an antibody class previously documented to internalize and induce target cell death [39] that binds SIINFEKL/H-2 Kb (see Additional file 1: Figure S4). Our data, overall, demonstrated an ability to generate and purify GrB_mut_-TRA that selectively facilitated target binding and cytotoxicity in vitro. Importantly, these properties were consistent with results obtained using the FDA-approved anti-HER2 ADC T-DM1.

GrB_mut_-TRA most closely models ADC platforms with site-specific attachment of payloads. Although the majority of clinically evaluated ADCs adopt random payload conjugations, site-specific attachment yields consistent product homogeneity and functional in vitro/in vivo activity [40] that are important hallmarks for ADC screening purposes. Given the need to activate GrB_mut_ with EK cleavage, we elected to engineer the GrB_mut_ payload to the N-terminus of TRA’s LC using a (G4S)3 linker to help minimize steric hindrance of the LC’s complementarity determining regions. Although alternate orientations may be possible, we did not observe substantial impediments to HER2 interaction that hindered binding and target cell cytotoxicity between GrB_mut_-TRA and T-DM1, but other antibody constructs could behave differently to the N-terminal placement of GrB_mut_ and impact properties such as target binding and AFP in vitro/in vivo stability. Regardless, we are not aware of other efforts that have designed a GrB-based AFP as reported. Relying on a non-immunogenic payload and conventional antibody framework (as opposed to other formats such as scFvs) not only mimics traditional and clinically validated ADC formats but also likely retains target avidity from a lead antibody candidate [41–43].

Our described ADC screening process may not easily lend itself to high-throughput examinations of antibody binders given the need to clone antibody VH and VL fragments into relevant vectors. However, with regard to VL placement, a backbone plasmid could be engineered in a manner to allow for easy adoption into a framework already containing GrB_mut_ and the constant LC domain that would facilitate a more robust pipeline for screening. It appears crucial though that an engineered variant of GrB be utilized. WT GrB contains a number of cationic sites that appear to non-specifically engage irrelevant proteins as reported by others [26–29] and further confirmed in this report (see Additional file 1: Figures S1-S2). Only after inducing relevant mutations at these suspected residues were we capable of reliably reducing off-target effects and distinguishing selective killing between cell lines deficient in or expressing HER2.

While GrB_mut_-TRA has only been evaluated in vitro, prior work has demonstrated the efficacy and therapeutic potential of disparate GrB-based antibody constructs in vivo [30, 33, 44–46]. Given our adoption of GrB_mut_ on a conventional antibody framework, we would anticipate an ability to safely treat and incite protection in tumor-bearing animals although future confirmatory work is required. Indeed, when GrB is conjugated to other constructs bearing an IgG Fc domain, PK in vivo appears stable [30]. In this manner, our described AFP would represent a compelling scaffold to expand pre-clinical assessments of ADC potential.

## Conclusions

Our current efforts provide proof-of-concept for an in vitro screening modality that incorporates a GrB_mut_-AFP to identify antibody candidates with desirable ADC properties. Such information would be invaluable to investigators prior to transitioning an antibody to the challenges/demands of ADC development and validation.

## Methods

### Cell lines and culture

The human breast adenocarcinoma SK-BR-3, human ovarian adenocarcinoma SK-OV-3, and murine melanoma B16-F10 cell lines (all from ATCC) were maintained in complete media containing RPMI 1640 (Cytiva) supplemented with 10% heat-inactivated FBS (Corning), penicillin/streptomycin/amphotericin B, 2 mM L-glutamine, 1 mM sodium pyruvate, and MEM non-essential amino acids (all from Thermo Fisher) at 37 °C with 5% CO_2_. B16-F10 engineered lines (designated B16.HER2 or B16.SII/Kb) were also created by stably expressing full-length HER2 (GenBank: M11730.1) or SIINFEKL/H-2 Kb using a Sleeping Beauty transposon plasmid system as previously described [47]. B16.HER2 and B16.SII/Kb cells were selected, clonally isolated, and maintained in complete media with 2 μg/mL puromycin. Use of parent/engineered B16 cell lines provided an improved evaluation of the effects of target expression prior to confirming in dissimilar cell lines naturally expressing the target. Cell lines were routinely screened and confirmed to be mycoplasma-free prior to in vitro studies.

### AFP expression and purification

Various mutations (R96A, R100A, R102A, R201K, C210A, K221A, K222A, K225A, R226A) were first introduced to wild-type (WT) human GrB (GenBank: M17016.1) (designated GrB_mut_) by splicing overlap extension PCR using the Phusion High-Fidelity DNA Polymerase (Thermo Fisher). A 6 × HIS-glycine-serine linker-FLAG tag was then added to the N-terminus of GrB_mut_ and the entire construct appended to the anti-SIINFEKL/H-2 Kb (SII) [48] or trastuzumab (TRA) variable light chain domain [49] (designated Pro-GrB_mut_-SII or Pro-GrB_mut_-TRA, respectively) as detailed in Fig. 1A.

In order to express full-length IgG antibodies, Pro-GrB_mut_-SII or Pro-Grb_mut_-TRA was cloned into the pRVL-4 plasmid (encoding the human kappa constant light chain domain) (a gift from Dr. Daniel Christ; Addgene plasmid # 104,582) using *SapI* restriction enzyme sites as described previously [50]. For control purposes, an unmodified version of the SII or TRA light chain domain was also incorporated in a separate pRVL-4 vector. The variable heavy chain sequence of SII [48] or TRA [49] was similarly cloned into the pRVL-5 plasmid (encoding the human IgG1 CH1/CH2/CH3 domains) (a gift from Dr. Daniel Christ; Addgene plasmid # 104,583).

Modified pRVL4/5 plasmids were transiently transfected into Expi293 cells using the Expi293 expression system according to the manufacturer’s recommendations (Thermo Fisher). Antibody from cell-free supernatant was then purified using HiTrap Protein G HP columns (Cytiva) with an ÄKTA start protein purification system (Cytiva). Eluted Pro-Grb_mut_-TRA IgG antibody was desalted in PBS using PD-10 columns (Cytiva) and enzymatically digested for 48 h at room temperature (RT) with His-tagged bovine enterokinase (EK) (GenScript) following the manufacturer’s instructions. The resulting antibody/enzyme mix was further purified by immobilized metal affinity chromatography (IMAC) using HisTrap excel columns (Cytiva). In this manner, the flow-through contained GrB_mut_-SII or GrB_mut_-TRA, whereas Pro-Grb_mut_-SII or Pro-Grb_mut_-TRA and EK were selectively bound by the column. Unmodified SII or TRA was purified by Protein G and desalted as indicated above. Ado-trastuzumab emtansine (T-DM1) (kindly provided under MTA by Genentech) was resuspended in PBS as detailed by the manufacturer. Final antibody preparations were 0.2 μM filtered and stored at 4 °C prior to use. Protein concentration was determined by UV absorbance at 280 nm.

### Western blotting

Purified antibody and recombinant human GrB (rGrB) (2906-SE, R&D Systems) were diluted in beta-mercaptoethanol-containing Laemmli SDS buffer (Bio-Rad), boiled, and resolved on a 4–15% gradient polyacrylamide gel (Bio-Rad) through SDS-PAGE. Coomassie blue staining was performed with PageBlue (Thermo Fisher) as indicated by the manufacturer. For western blotting, proteins were transferred to PVDF membrane (Millipore-Sigma), blocked with 5% non-fat dry milk for 1 h at RT, and incubated overnight at 4 °C with an appropriate antibody: anti-FLAG (L5, Biolegend), anti-human granzyme B (AF2906, R&D Systems), or peroxidase-anti-human IgG (709–035-149, Jackson ImmunoResearch). Blots were washed with 0.5% Tween 20 in PBS (PBST) and probed with either goat anti-rat IgG-HRP (A10549, Thermo Fisher) or bovine anti-goat IgG-HRP (805–035-180, Jackson ImmunoResearch) reagents for 1 h at RT. Following additional wash steps, blots were developed using SignalFire ECL (Cell Signaling Technology) and imaged on a ChemiDoc Touch imaging System (Bio-Rad).

### ELISA

Recombinant HER2 (10,004-HCCH, Sino Biological) or irrelevant protein was immobilized in triplicate on ELISA plates (Thermo Fisher) at 2 μg/mL in 100 μL of PBS overnight at 4 °C. Following consecutive washes with PBST, plates were blocked with 10% FBS/PBS for 1 h at RT, washed, and incubated with 100 μL aliquots of serial dilutions of antibodies of interest at RT for 2 h. Following additional wash steps in PBST, an HRP-conjugated donkey anti-human reagent (709–035-149, Jackson ImmunoResearch) in blocking buffer was applied for 1 h at RT. Plates were washed, developed using a 1-Step Ultra TMB reagent (Thermo Fisher Scientific), and enzymatic reactions stopped with an HCL-based solution (SeraCare). Plates were then immediately measured by absorbance at 450 nm using a BioTek Cytation 5 Reader.

### GrB activity

GrB-specific enzyme activity was measured using the colorimetric substrate Ac-IEPD-pNA (Enzo Life Sciences) (a substrate for GrB and caspase-8) in assay buffer (100 mM Tris, 200 mM NaCl, 0.01% Tween 20, pH 7.4) as demonstrated previously [51]. rGrB was also incorporated as a control after being activated with mouse Cathepsin C (R&D Systems). Various concentrations of GrB-based antibody constructs and molar equivalent concentrations of rGrB were added in 90 μL aliquots to a 96-well plate in triplicate, covered, and incubated at 37 °C for 30 min. Ac-IEPD-pNA (1.25 mM in assay buffer) was then introduced in 10 μL aliquots to all wells and immediately transferred to a climate-controlled BioTek Cytation 5 Reader at 37 °C for kinetic absorbance measurements at 405 nm every 5 min up to 1 h.

### Immunofluorescence (IF)

B16-F10 and B16.HER2 cells were added in complete media to an 8-well chamber slide (5 × 10^4^ cells/chamber) (Thermo Fisher) for 4 h at 37 °C. Chambers were then treated with activated rGrB, TRA, or GrB_mut_-TRA at molar equivalent concentrations for 1 h. Media was aspirated and cells were exposed to an acid wash (500 mM NaCl, 100 mM glycine, pH 2.5) for 5 min and then neutralized with 500 mM Tris at pH 7.4. Cells were then fixed in 4% formaldehyde in PBS for 10 min, washed with PBS, and permeabilized using 0.2% Triton X-100 for 10 min. Following PBS washes, cells were blocked with 3% BSA, incubated with an anti-human granzyme B antibody (AF2906, R&D Systems) in blocking solution for 1 h, washed with PBST, and exposed to a secondary anti-goat or human AF488 reagent (805–545-180, 709–545-149 Jackson ImmunoResearch) for 1 h. Coverslips were applied over an anti-fade mounting medium containing DAPI (Vector Laboratories) and processed for IF microscopy using a MICA Microhub microscope (Leica).

### Flow cytometry

Cell lines were resuspended in FACS buffer (0.5% BSA/0.1% NaN_3_ in PBS) and incubated with various antibody constructs at 2 μg/mL for 20 min at 4 °C. hIgG1 binding to target cells was assessed using a PE-conjugated goat anti-human antibody (12–4998-82, Thermo Fisher). Pro- and mature- forms of GrB-TRA bound constructs were also detected using a mouse anti-His tag antibody (652,502, Biolegend) followed by a PE-conjugated goat anti-mouse reagent (115–115-164, Jackson ImmunoResearch).

To determine caspase 3/7 activity, 3 × 10^5^ target cells were seeded in complete media in a 6-well plate and cultured for 8 h. Non-adherent cells and media were removed and replaced with fresh complete media containing antibody constructs at various concentrations to generate proportionate, non-total cytotoxic responses. Following overnight incubation at 37 °C with 5% CO_2_, cell media and adherent cells were collected and incubated with Cell Event Caspase-3/7 Green Detection Reagent and SYTOX AADvanced Dead Cell Stain (Thermo Fisher) per the manufacturer’s instructions.

Flow cytometric analysis was performed on cells using a BD LSR Fortessa (BD Biosciences) and FlowJo v 10.8.1 software (BD Life Sciences).

### In vitro cytotoxicity

Select tumor cells (4 × 10^3^ cells/well) were seeded in triplicate in complete media in flat bottom 96-well plates overnight at 37 °C with 5% CO_2_. Media was aspirated, replaced with fresh complete media containing serially diluted concentrations of antibody constructs, and incubated for another 48 h. Cell viability was then determined using a modified crystal violet staining protocol. Briefly, aspirated wells were fixed with a 2% paraformaldehyde/2.5% glutaraldehyde solution (Electron Microscopy) for 30 min, washed with water, and stained with 0.05% crystal violet/20% ethanol for 30 min at RT. Plates were washed three times with water and air dried for 1 h. The crystal violet stain was next solubilized in 100 μl 10% acetic acid under orbital shaking for 20 min at RT. Plates were read by absorbance at 570 nm using a BioTek Cytation 5 Reader. Target cell viability was calculated based on signals obtained from non-treated control wells and cells exposed to 10 μM camptothecin that ensured maximum cell death.

### Statistical analysis

Select results were analyzed with one-way ANOVA + post hoc pairwise comparisons using GraphPad Prism (version 9.3.1). Mean differences with a *P* value < 0.05 were considered statistically significant. Data presented are representative of at least 3 independent experiments.

### Supplementary Information

**Additional file 1: Figure S1.** GrB-TRA non-specific binding and cell cytotoxicity. **Figure S2.** Additive interference of WT GrB non-specific binding. **Figure S3.** Demonstration of pro-peptide cleavage following GrBmut-TRA EK processing and purification. **Figure S4.** GrBmut-SII selectively binds its target and induces cytotoxicity in vitro against cells expressing SIINFEKL/H-2 Kb. **Figure S5.** Original gel/western blots corresponding to the cropped gel/western blots in Fig. 1. **Figure S6.** Original western blot corresponding to the cropped western blot in Figure S3.

**Additional file 2. **Individual values for data analyzed in this study.

## Data Availability

All data generated or analyzed during this study are included in this published article and its supplementary information (see “Additional file 2”).

## Abbreviations

ADC: Antibody drug conjugates
AFP: Antibody fusion protein
EK: Enterokinase
GrB: Granzyme B
GrB_mut_: Mutated GrB
rGrB: Recombinant GrB
HC: Heavy chain
IF: Immunofluorescence
IMAC: Immobilized metal affinity chromatography
LC: Light chain
PBST: 0.5% Tween 20 in PBS
RT: Room temperature
SII: SIINFEKL/H-2 Kb
T-DM1: Ado-trastuzumab emtansine
TRA: Trastuzumab
WT: Wild-type
