# Supplementary material for: Modeling antibody drug conjugate potential using a granzyme B antibody fusion protein
Source: BMC Biol. 2024 Mar 14;22:66. doi: 10.1186/s12915-024-01860-x (PMC10941411; doi:10.1186/s12915-024-01860-x)
Supplement: Supplementary file 1 — Additional file 1: Figure S1. GrB-TRA non-specific binding and cell cytotoxicity. Figure S2. Additive interference of WT GrB non-specific binding. Figure S3. Demonstration of pro-peptide cleavage following GrBmut-TRA EK processing and purification. Figure S4. GrBmut-SII selectively binds its target and induces cytotoxicity in vitro against cells expressing SIINFEKL/H-2 Kb. Figure S5. Original gel/western blots corresponding to the cropped gel/western blots in Fig. 1. Figure S6. Original western blot corresponding to the cropped western blot in Figure S3. [file 12915_2024_1860_MOESM1_ESM.docx]

**Additional File 1. Figures S1-S6**

**
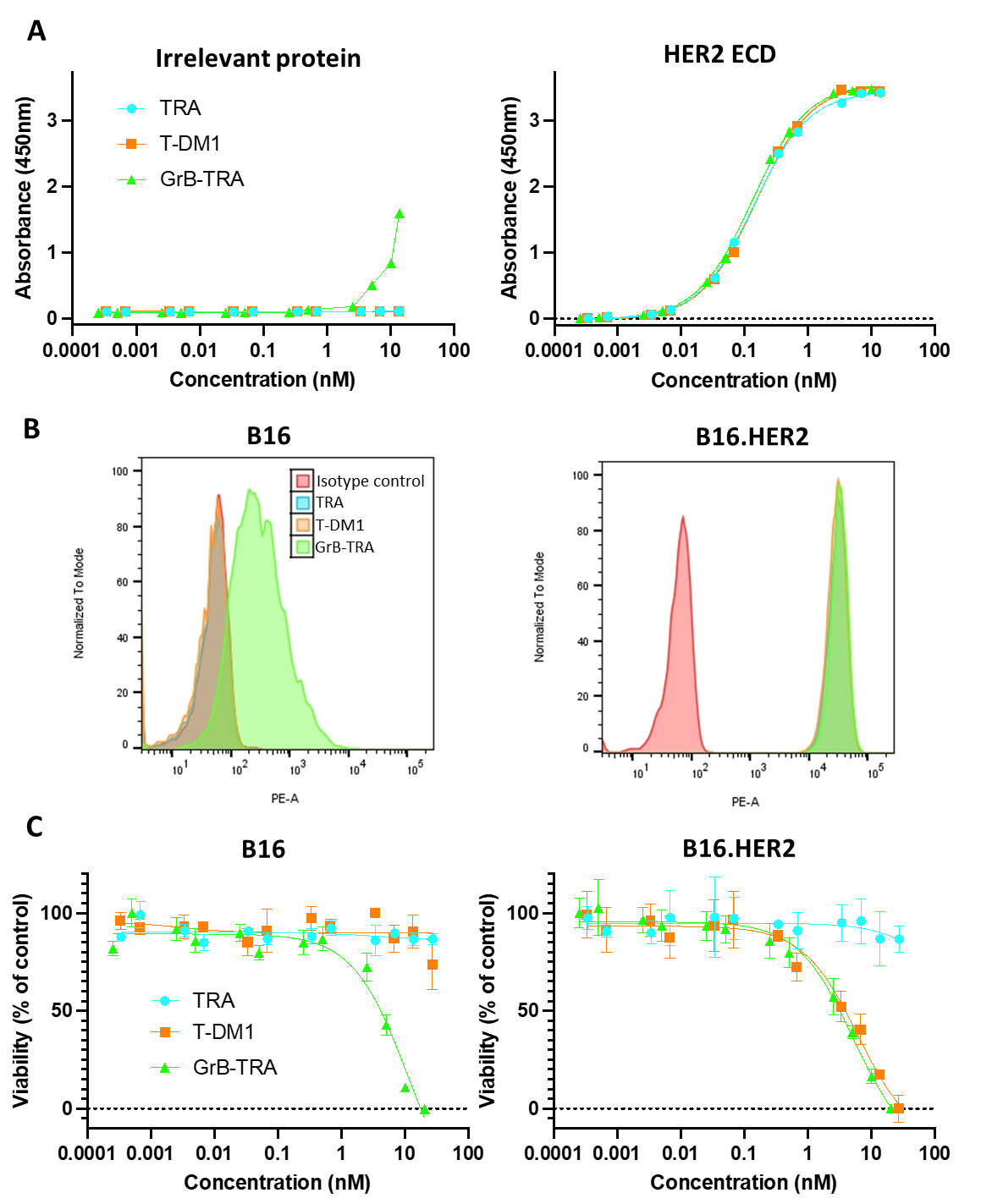
**

**Figure S1:** GrB-TRA non-specific binding and cell cytotoxicity. (A) TRA, T-DM1, and GrB-TRA binding properties against an irrelevant protein or HER2 ECD at various concentrations as determined by ELISA. (B) Confirmation of target binding through flow cytometry using B16 and B16.HER2 cell lines. (C) Target cell viability plots (based on crystal violet staining) following 48 hr exposure to TRA, T-DM1, or GrB-TRA. Abbreviations used: ECD, extracellular domain; GrB, granzyme B; rGrB, recombinant GrB; GrB-TRA, catalytically active AFP with WT GrB; T-DM1, ado-trastuzumab emtansine; TRA, trastuzumab. Bars ± STDEV. Select results are based on technical replicates of 3 samples per treatment group. Individual data values are provided in supplementary information (Additional file 2).


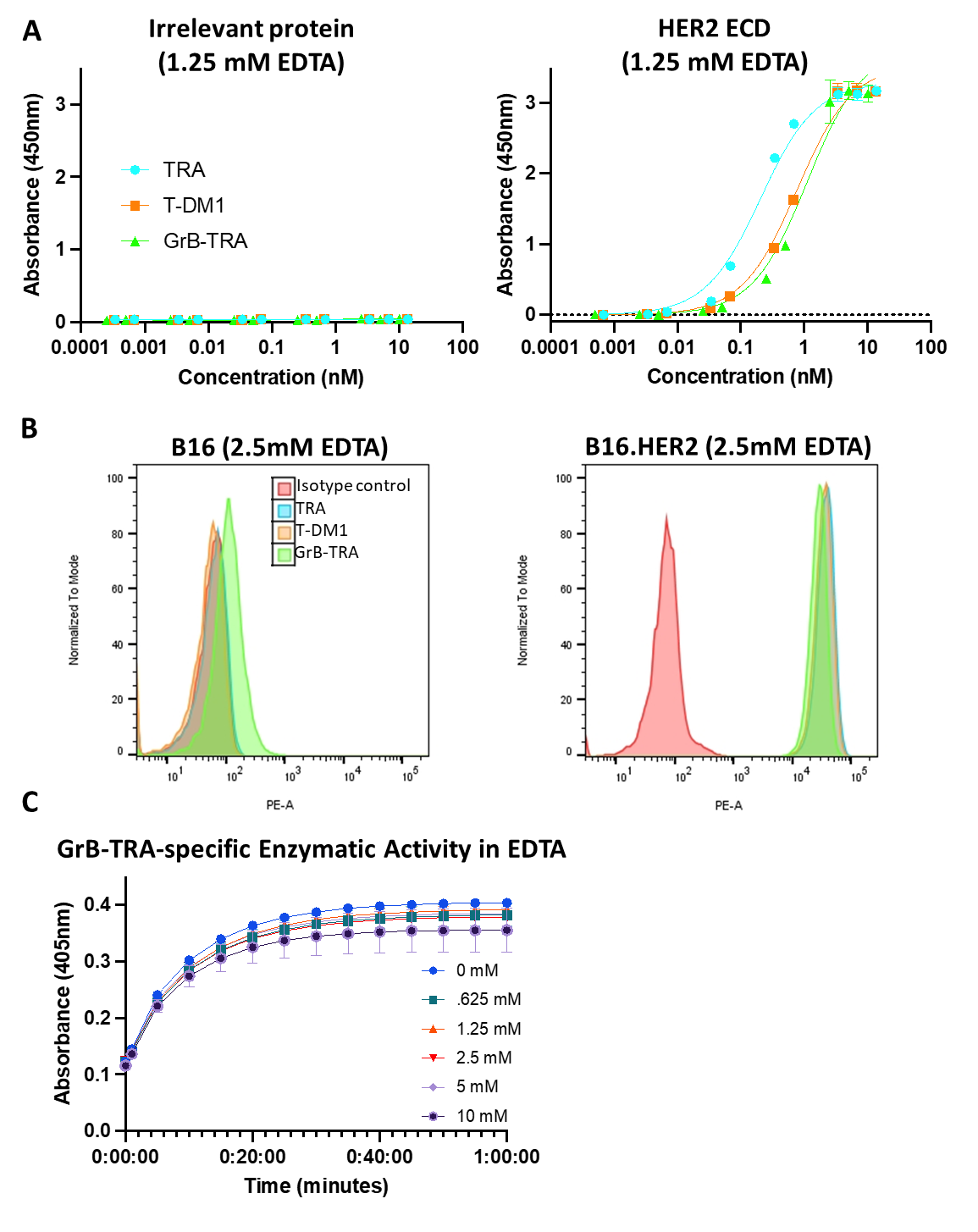


**Figure S2:** Additive interference of WT GrB non-specific binding. EDTA was incorporated to minimize off-target binding effects of WT GrB as determined by (A) ELISA and (B) flow cytometry. (C) The functional activity of WT GrB (conjugated to TRA) was subsequently assessed by absorbance using a chromogenic Ac-IEPD-pNA substrate in the presence of various concentrations of EDTA. Bars ± STDEV. Select results are based on technical replicates of 3 samples per treatment group. Individual data values are provided in supplementary information (Additional file 2).


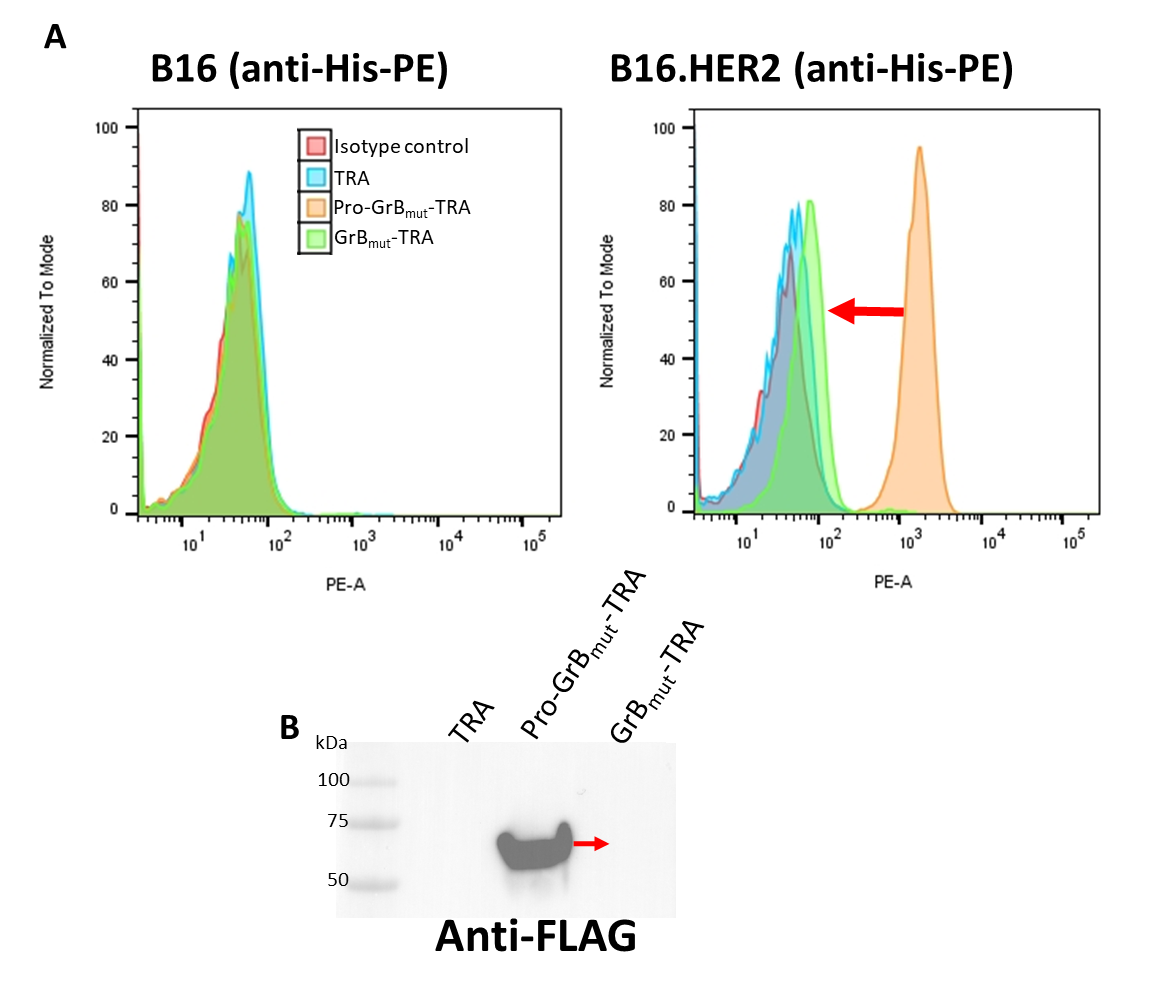


**Figure S3:** Demonstration of pro-peptide cleavage following GrBmut-TRA EK processing and purification. To generate a catalytically active and purified AFP, the N-terminal pro-peptide (incorporating 6x HIS-GGS-FLAG tag) of GrBmut was cleaved by EK and subjected to IMAC. (A) The integrity of this process was assessed by probing for the 6x HIS tag on the AFP’s pro-peptide using AFP constructs that bound B16.HER2 cells by flow cytometry. (B) Western blot was also incorporated to probe for the FLAG tag of the pro-peptide in purified fractions of TRA, Pro-GrBmut-TRA, and GrBmut-TRA. Arrow insets demonstrate the relative absence of the 6x HIS and FLAG tags in GrBmut-TRA. Abbreviations used: EK, enterokinase; GrB, granzyme B; GrBmut-TRA, catalytically active AFP; Pro-GrBmut-TRA, catalytically inert molecule; R, TRA, trastuzumab.


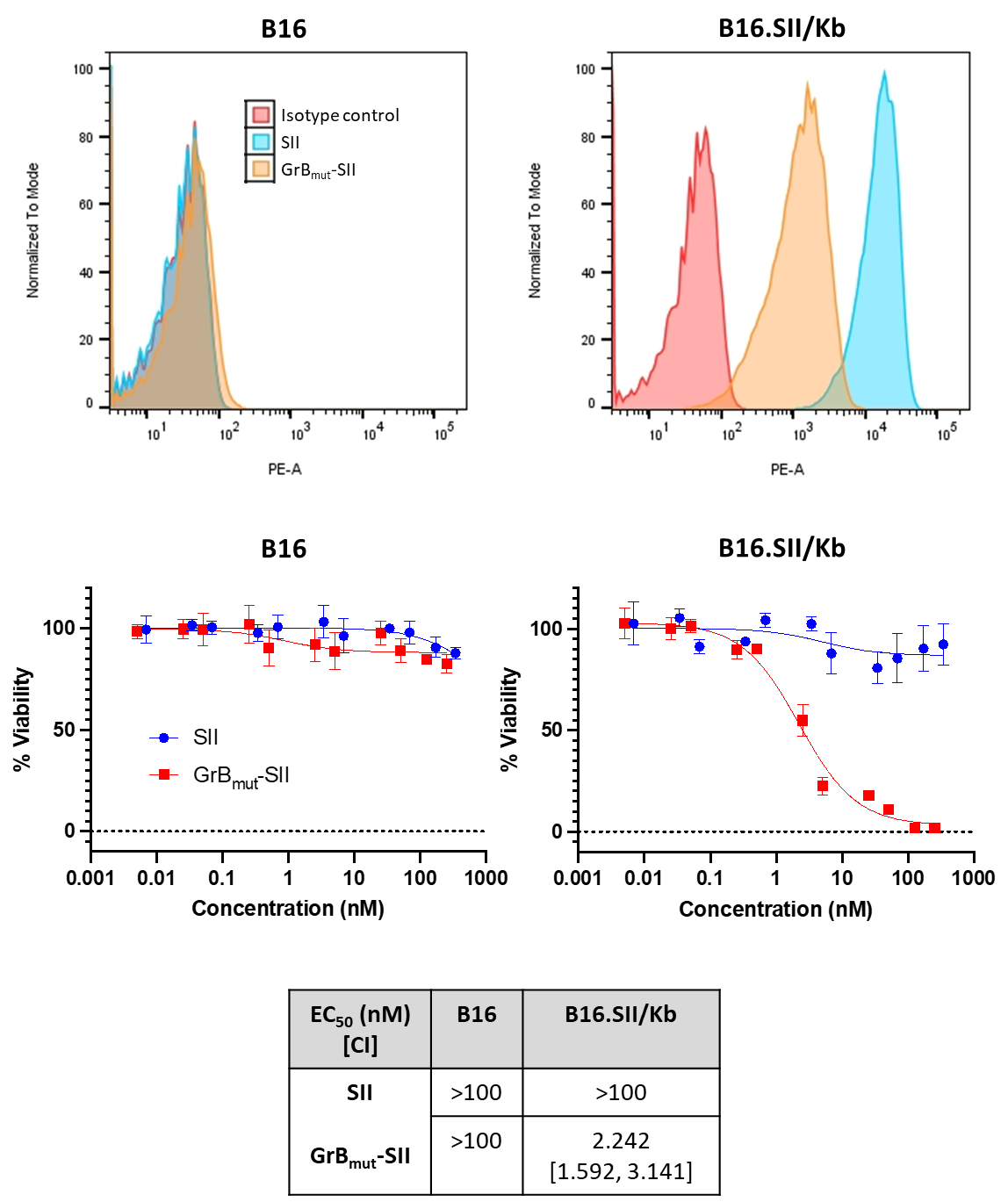


**Figure S4:** GrBmut-SII selectively binds its target and induces cytotoxicity in vitro against cells expressing SIINFEKL/H-2Kb. A separate AFP was generated against the SIINFEKL/H-2Kb molecule (designated GrBmut-SII). Similar to experimental details in Figures 2 and 4, target binding and cell cytotoxicity were assessed using B16 cells expressing/not expressing SIINFEKL/H-2Kb through flow cytometry and crystal violet staining, respectively. Abbreviations used: B16.SII/Kb, B16 cells engineered to express SIINFEKL/H-2Kb; CI, 95% confidence intervals; GrB, granzyme B; GrBmut-SII, catalytically active AFP; SII, anti-SIINFEKL/H-2Kb parent antibody. Bars ± STDEV. Select results are based on technical replicates of 3 samples per treatment group. Individual data values are provided in supplementary information (Additional file 2).


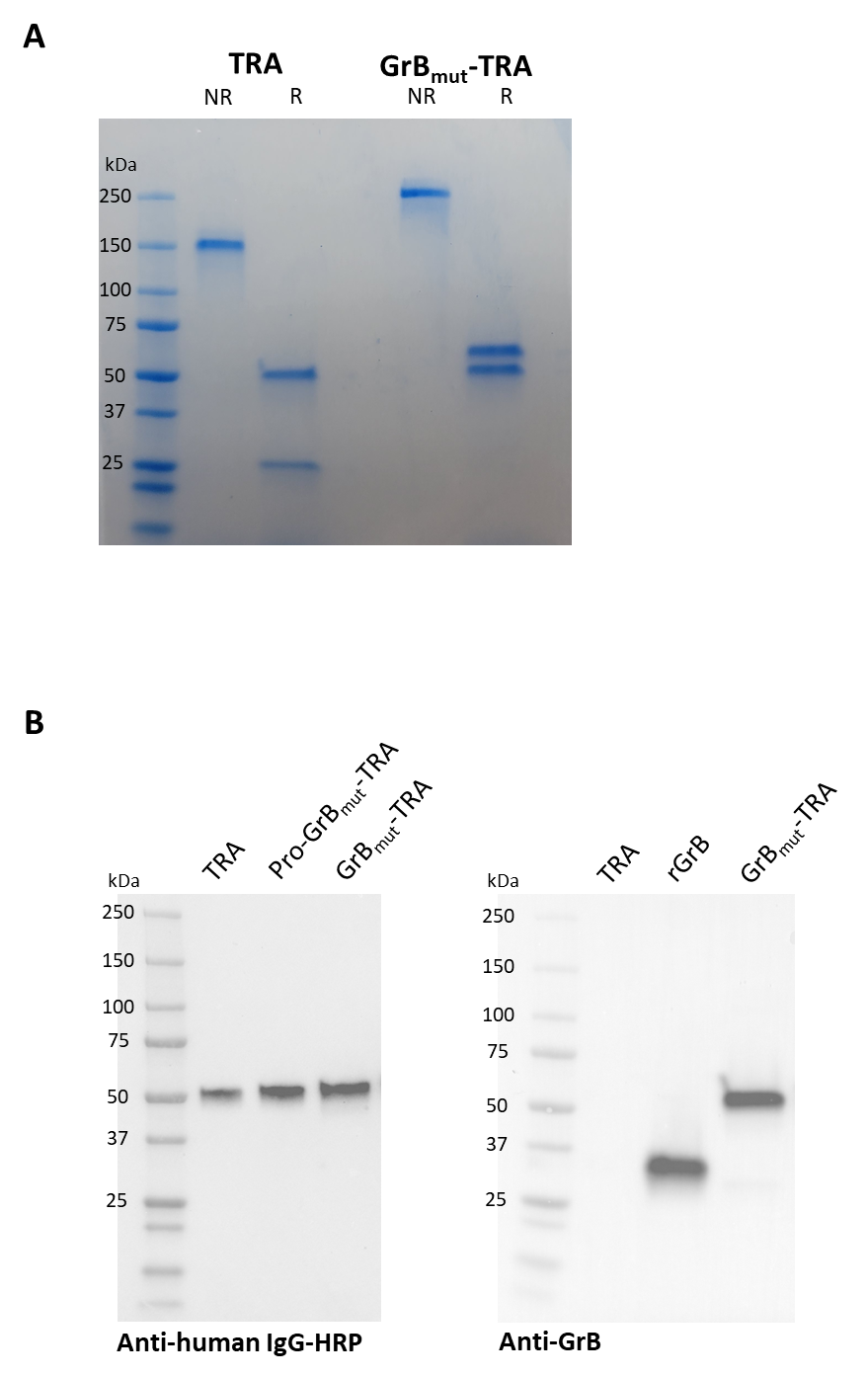


**Figure S5.** Original gel/western blots corresponding to the cropped gel/western blots in Figure 1.


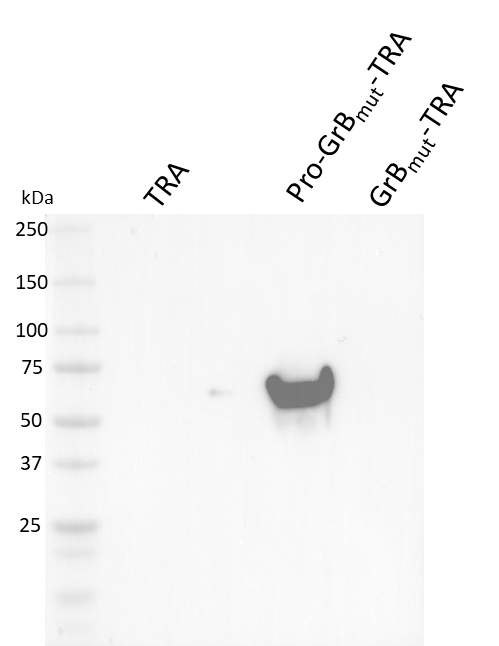


**Figure S6.** Original western blot corresponding to the cropped western blot in Figure S3.
